# Beyond prescriptions: chronic medication adherence predicts mortality risk in a large-scale cohort study

**DOI:** 10.3389/fphar.2025.1701588

**Published:** 2025-11-25

**Authors:** Jessica Hamuy Blanco, Dina C. Janse van Rensburg, Audrey Jansen van Rensburg, Corrie Uys, Natalie Schellack

**Affiliations:** 1 Department of Pharmacology, Faculty of Health Sciences, University of Pretoria, Pretoria, South Africa; 2 Section Sports Medicine and SEMLI, Faculty of Health Sciences, University of Pretoria, Pretoria, South Africa; 3 Applied Microbial and Health Biotechnology Institute, Cape Peninsula University of Technology, Cape Town, South Africa

**Keywords:** medication adherence, medication compliance, medication persistence, drug persistence, chronic medication, mortality, death, survival rate

## Abstract

**Objectives:**

The Medication Adherence Risk Score (MARS) is a calculated score using pharmacy transactional data spanning 50% of the South African private pharmacy market. This study aims to demonstrate that the existing MARS model enhances risk stratification by identifying individuals at increased risk of mortality related to non-adherence to chronic medication.

**Methods:**

This was a retrospective cohort study in which an analysis of the relative mortality experience was compared to a standard fully underwritten base was performed for each of the MARS categories (low, medium, high and very high). The actual-to-expected ratio (AER) and relative risk (RR) for each category were compared across age groups and gender. The least absolute shrinkage and selection operator (LASSO) regression analysis method was applied to determine the most important variables within the dataset, providing insight into whether MARS offered more benefit than traditional risk rating factors. A time-to-event analysis by MARS categories was performed using the Cox proportional hazards model.

**Results:**

The mortality experience of the study population was higher than the expected fully underwritten base (AER = 175%). For the overall sample, increasing AER and RR did not correlate with increasing MARS categories. However, use of the MARS in addition to age band allowed for differentiation of risk within the 25 to 55 age bands, with a higher MARS score indicating a higher AER and RR. The time-to-event analysis showed a statistically significant difference in the mean number of months before death occurred between the different MARS categories (low = 26.53; medium = 8.93; high = 7.02; very high = 6.92; p < 0.001).

**Conclusion:**

The MARS is not generalisable across all groups, as evidenced by the absence of a monotonic trend in the overall sample. However, when combined with age, it effectively differentiated mortality risk for individuals aged 25–55. The standard fully underwritten model underestimated the number of deaths within this pharmacy population. The time-to-event analysis showed a significant inverse relationship between MARS category and survival time.

## 1 Introduction

Seven of the top ten natural causes of death in South Africa (diabetes mellitus, cerebrovascular diseases, heart disease, human immunodeficiency virus (HIV), hypertensive diseases, ischaemic heart disease and chronic lower respiratory diseases) (Africa DoSS, 2018) are chronic conditions with established pharmacological treatments. South Africa is uniquely impacted due to its multiple burdens of disease, characterised by high rates of infectious diseases such as HIV and tuberculosis, alongside non-communicable diseases such as cardiovascular disease and diabetes (Modjadji, 2021). Two or more active diseases are prevalent in approximately 11.8% of the population (Wong et al., 2021). The management of multi-morbidity is more complex and demanding for health systems and patients (Boyd and Fortin, 2010). Individuals with multiple chronic conditions are more likely to experience lower quality of life, disability, premature mortality, greater use of healthcare services, and increased number of hospital admissions (Doessing and Burau, 2015).

Deviation from the intended dosage and frequency of medication use may limit the therapeutic effect and allow for increased disease progression (Khunti et al., 2017; Du et al., 2017). A recent systematic review (Hamuy Blanco et al., 2025) showed a clear association between lower adherence rates and higher risk of mortality. Poor adherence and non-adherence had the highest HRs (1.63 and 2.77, respectively). This relationship was consistent across the varying disease areas, adherence metrics and whether all-cause or specific causes of mortality were investigated. Poor medication adherence contributes to suboptimal chronic disease management, leading to significant increases in the cost of healthcare (Chauke et al., 2022; Elliot, 2009).

While many studies demonstrate a correlation between poor medication adherence and mortality (Kim et al., 2018; Kim et al., 2016; Asher et al., 2012), the use of medication adherence rates in assessing clinical risk has been limited by challenges regarding its assessment and interpretation. The use of different formulae for calculating adherence rates (Baumgartner et al., 2018) makes it challenging to compare results. The threshold to distinguish between adherent and non-adherent individuals (typically 80%) (Baumgartner et al., 2018) has seldom been linked to clinical effects and has recently been questioned (Baumgartner et al., 2018; Stauffer et al., 2017). Furthermore, patients and healthcare providers overestimate adherence when providing a subjective rating of the problem (Schaefer et al., 2019; Shi et al., 2010). Given the difficulties with assessing and interpreting medication adherence measurements, the use of such a metric in decision making has been limited (Fitzgerald and Ryan, 2019).

The Medication Adherence Risk Score (MARS) is a calculated score using pharmacy transactional data obtained from Health Window (a registered company specialising in managing chronic medication adherence). Electronic pharmacy records have been used to measure adherence in international studies (Sun et al., 2024; Amiesimaka et al., 2024; Galozy et al., 2020), and in some instances with a view to understand its relationship to mortality risk (Bailey et al., 2010; Fo et al., 2019; Wong et al., 2013). At the time of the study, Health Window partnered with pharmacies comprising roughly 50% of the private South African market. It received daily feeds of pharmacy transactional data in order to deliver the medication adherence service. This data was processed using various metrics, including a chronic medication adherence score (CMAS) and the MARS. The CMAS is a form of the widely recognised medication possession ratio, representing the proportion of doses dispensed out of doses prescribed, over a defined time period (Stauffer et al., 2017). The CMAS may have some inherent limitations in predicting mortality risk. The MARS considers the number of chronic diseases, the duration of treatment, and the variability in risk across different conditions, whereas the CMAS does not.

An objective and reliable adherence-based score, such as MARS, available for a large portion of the South African population, would contribute to an improved and more individualised risk evaluation. This could lead to favourable commercial structures for consumers adhering to their treatment. For example, during health risk assessment when taking out a life insurance policy, individuals diagnosed with the same chronic condition receive a similar risk rating, irrespective of the extent of their treatment adherence (Fitzgerald and Ryan, 2019). Furthermore, non-adherence contributes to higher claims on private medical insurance products, increasing premiums to cover aggregate risks (Fitzgerald and Ryan, 2019). In future, practical measures to incorporate medication adherence into the prediction of outcomes could play a role in the move toward value-based reimbursement structures for healthcare providers (van Staalduinen et al., 2022). Further applications may include adherence scores in doctor-patient consultations and pre-operative evaluations. This study aims to demonstrate that the existing MARS model can enhance risk stratification efforts by identifying individuals at increased risk of mortality related to non-adherence to chronic medication.

## 2 Materials and methods

A retrospective cohort design was used to conduct this research.

### 2.1 Data and data collection

Data was sourced from Health Window–a registered company specialising in managing chronic medication adherence and, at the time of the study, partnering with pharmacies comprising roughly 50% of the private South African market. Health Window received daily feeds of pharmacy transactional data in order to deliver the service. This data included information on customer demographics (South African ID number, date of birth, age, name, surname, gender, address), medical aid (medical scheme number, scheme option, dependant code), medication dispensed (product name, nappi code, dose, quantity dispensed, manufacturer, day’s supply, instructions, original repeats, repeats left), prescribing doctor (practice number, prescriber name) and pharmacy (store name, rams number, address). Health Window imports this data and matches the following additional fields to each product: Monthly Index of Medical Specialties (MIMS) classes 1 to 3, Anatomical Therapeutic Chemical (ATC) level 1 to 5, and product classification (acute, chronic or could be chronic).

### 2.2 Health Window metrics

Health Window calculated the CMAS and MARS from the transactional data. Table 1 shows details of these metrics. A particular individual would be allocated a CMAS and/or MARS for a particular month, provided that their historical data included transactions within a partner pharmacy far enough into the past and that they had purchased medication from a relevant class. In instances where these criteria were not met, an “unknown” category would be assigned for that month. This meant that individuals assigned to the “unknown” category had no observable presence of chronic disease and were likely healthier, with sporadic pharmacy use. Supplementary Material 1 provides further explanation of the CMAS and the MARS.

**TABLE 1:** Health Window metrics.

Health Window metric	Description	Risk categories	Risk score range
CMAS	A calculated score based on the frequency of chronic medication dispenses over a year. This is a measure of the MPR.	Very poor	<50%
		Poor	50%–65%
		Fair	65%–80%
		Good	>80%
MARS	A calculated score following information obtained from the pharmacy transactional data 1. Number of unique risk-labelled MIMS classes from which a chronic product has been purchased at least 3 times in the last 24 months 2. CMAS for each applicable MIMS class 3. Duration of use of treatment within each MIMS class	Very high	Top 1% of health window population
		High	3.05 ≤ *x* < lower limit of very high population
		Medium	0.11 ≤ *x* < 3.05
		Low	*x* < 0.11

CMAS: chronic medication adherence score.

MARS: medication adherence risk score.

MIMS: monthly index of medical specialties.

MPR: medication possession ratio.

### 2.3 Study population and sampling

The study population is 9,125,862 unique patient cases within the dataset of dispensary transactions from Health Window network pharmacies. The study was limited to individuals with transactions during the exposure and outcome measurement periods. Supplementary Material 2 further explains the criteria used for sample inclusion and exclusion.

### 2.4 Data deidentification and matching with mortality indicator

Three data tables were compiled containing the following information on the Health Window patient population: patient demographics (South African ID number, name, surname, date of birth, cell phone number, age, gender), monthly rating of CMAS per patient case for the period 1 January 2017 to 1 May 2022, monthly MARS per patient case for the period 1 January 2018 to 1 May 2022. The MARS calculation requires a 1-year history of chronic adherence scores, resulting in a 1-year lag between the time periods.

These data tables were uploaded to the Omnisient Privacy-Preserving Data Collaboration Platform, a registered company’s technology that matches records across multiple datasets while adhering to global consumer privacy regulations. Upon upload, all personally identifiable information was anonymised on-premises to create irreversible “Crypto-identities,” assigning each individual patient case a new unique “Crypto-identifier.” This allows for identifying the same individual within other datasets on the platform without being traced back to any identifiable data. By utilising the Omnisient platform, the study could leverage a secure and efficient data management system that protected consumer data while facilitating collaboration and generating valuable insights.

An overlap analysis was conducted between the patient cases in the Health Window datasets and an existing dataset on the Omnisient platform containing a mortality indicator and date of death for individuals who have passed away. The number of individuals within both datasets was 254,810. The de-identified datasets, including the mortality dataset, were then returned to Health Window. An analysis of the expected number of deaths in each MARS group (according to standard risk rating methods) was performed by a reinsurance company with which Health Window has a working relationship.

### 2.5 Data analysis

The data was cleaned to exclude patient cases that did not meet at least one inclusion criteria or met any exclusion criteria (Supplementary Material 2). Exposure measures were defined by the duration of time that an individual possesses a certain factor within the data, e.g., the time within a particular age range, or the time within a particular MARS category. For example, an individual with a “high” MARS for 12 months, would contribute exposure to the high MARS category for those 12 months. These exposure measures were linked to the outcome measure. An expected basis (number of deaths expected from a standard fully underwritten model) was assigned to the MARS groups.

Due to the billing and regulatory requirements in the private pharmacy market, completeness of patient records is paramount. There are a small number of instances where data may be missing or incorrectly captured. An incorrect date of birth or identity number could result in a patient’s age being reflected as a negative number or a blank field. These cases made up 0.2% of the total records and were excluded from the sample. An identity number containing too few digits, too many digits or text characters prevented the researchers from correctly matching an individual to the mortality indicators provided by Omnisient. This occurred in less than 1% of all records.

A standard fully underwritten model is the traditional approach in life insurance, where each client undergoes a detailed risk assessment before being offered coverage (Consultants TSAa, 2022). Insurers gather comprehensive information about the client’s age, gender, health, lifestyle, occupation, education and financial circumstances to assess their overall mortality and morbidity risk. This often includes detailed questions about the client’s past and current health status. Depending on the client’s specific details and risk assessment, additional medical examinations (e.g., pathology and diagnostic tests) may be required to assess the risk profile accurately. Certain disclosures made by the client may warrant issuing of further questionnaires to obtain a detailed understanding of the conditions noted. Beyond the information on the application, insurers may also rely on independent sources, e.g., previous pathology results, doctors’ reports, claims summaries and financial documentation. These are used to verify disclosures as well as to build a more complete risk profile. The underwriting decision is made by a risk expert who integrates all evidence and categorises the applicant’s risk appropriately. Thereafter, the client is offered an insurance premium and cover amount that corresponds to their overall risk.

A standard fully underwritten insurance cohort would typically have a better risk profile compared to the general population, or to cohorts that have not undergone underwriting. There is an element of selection bias, as the underwriting process will decline or charge higher premiums to individuals with higher risk profiles, meaning that they self-select out of the pool. This cohort was included to offer a baseline for comparison. It serves as a valuable benchmark for risk analysis because it is designed to account for as many risk factors as possible. Therefore, comparing another cohort to a fully underwritten base allows for a clearer understanding of relative risk.

An analysis of the relative mortality experience was performed to assess the difference across the various categories of the MARS. The relative experience is the expected number of deaths based on typical risk rating factors, compared to what has actually occurred, i.e., the actual-to-expected ratio (AER). The relative risk (RR) is calculated by dividing the AER for a particular factor by the overall AER. RR is 100% when the number of actual deaths equals the number of expected deaths. The least absolute shrinkage and selection operator (LASSO) regression analysis method was applied to determine the most important variables within the dataset, providing insight into whether the use of MARS offered more benefit than traditional risk rating factors. The co-variates included in the model were age band, gender and MARS category.

A time-to-event analysis by MARS categories was performed using the Cox proportional hazards model, comparing the mean time (in months) to death across the different MARS categories. Schoenfeld plots were used to test the proportional hazards assumption, which was not violated.

## 3 Results

There were 7,598,885 individuals with a history of purchasing chronic medication during the study period (prevalence of 83.33%). The overall AER using the fully underwritten mortality basis was 175%, and the overall crude rate per million was 5.48.

**FIGURE 1:**
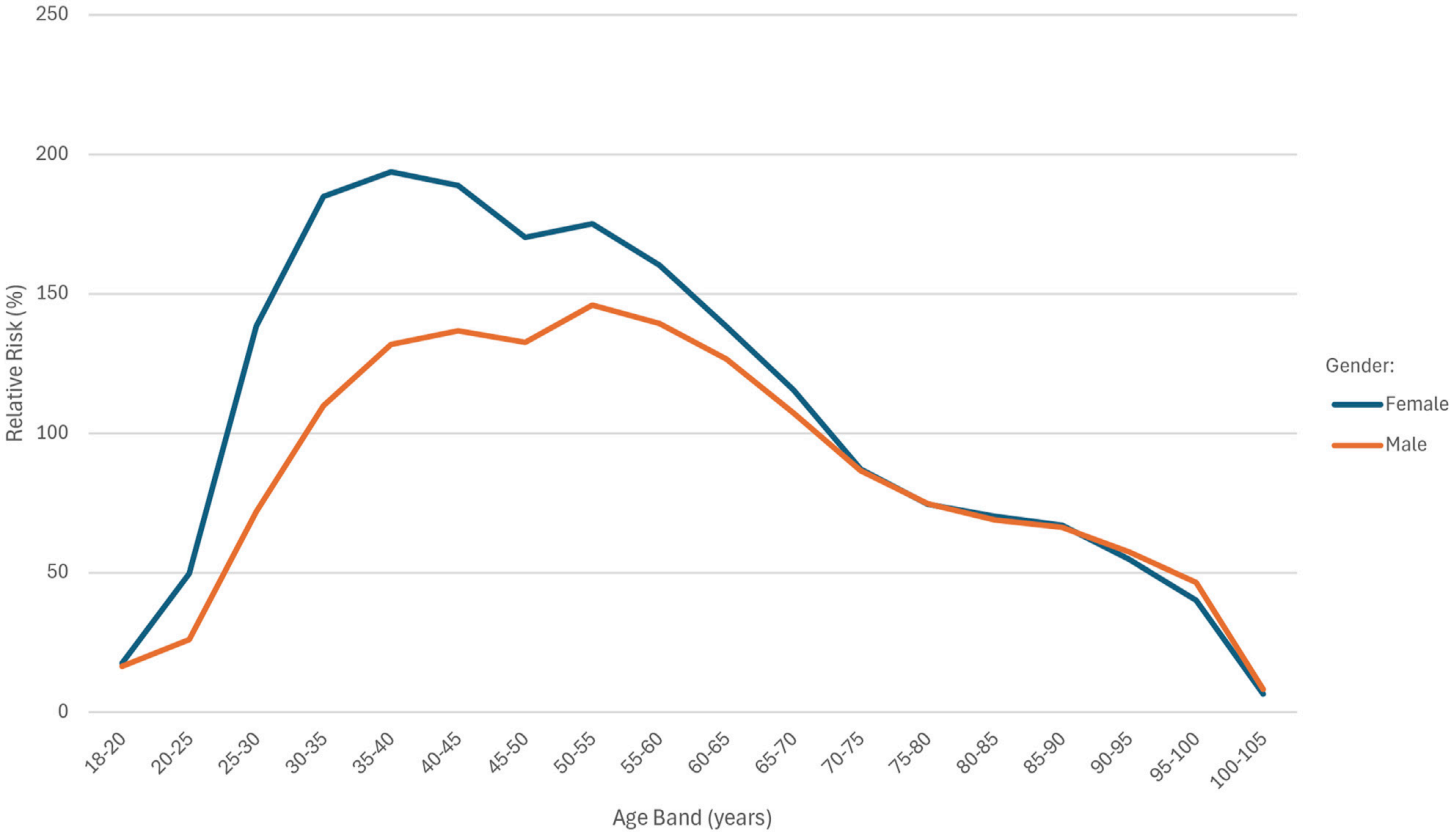
RR by age band and gender.

Table 2 shows the AER and RR by age band. Figure 1 depicts the AER and RR by age band and split by gender.

**TABLE 2:** AER and RR by age band.

Age band	Exposure	Number of deaths	AER (%)	RR (%)	Crude rate per million
18–20	372,714	42	29.14	16.65	0.1127
20–25	1,970 190	503	56.52	32.3	0.2553
25–30	3,346 093	2,237	162.44	92.82	0.6685
30–35	4,375 207	5,093	237.98	135.99	1.1641
35–40	4,604 111	8,036	270.55	154.6	1.7454
40–45	4,029,790	9,998	273.52	156.3	2.4810
45–50	3,817 470	12,953	257.08	146.9	3.3931
50–55	3,487 332	18,301	274.22	156.7	5.2479
55–60	2,734 661	20,295	257.33	147.05	7.4214
60–65	2,193,916	21,887	229.19	130.97	9.9762
65–70	1,571 726	20,499	193.41	110.52	13.0423
70–75	1,122,834	18,698	151.7	86.68	16.6525
75–80	749,856	17,192	130.37	74.5	22.9271
80–85	462,589	15,549	121.55	69.46	33.6130
85–90	231,676	12,211	116.46	66.55	52.7072
90–95	93,531	7,239	97.45	55.69	77.3968
95–100	21,204	2,087	73.57	42.04	98.4248
100–105	1,043	27	12.31	7.03	25.8869

AER, actual-to-expected ratio.

RR, relative risk.

Exposure was relatively low in the 18–20 and 80–105 age bands, with the highest exposure in the 35–40 age band. The highest number of deaths occurred in the 60–65 age band. The AER values were below 100% in the 18–25 and 90–105 age bands, while AER exceeded 200% in the 30–65 age range. A RR of above 100% was observed in the 30 and 70-year age groups. The highest crude rate (98.4248) was recorded in the 95–100 age band.

If the effect pattern of age in the fully underwritten basis matched that of the study population, the RR across age bands would be represented by a horizontal line. In Figure 1, the age distribution curves for both males and females deviate from a horizontal line, indicating age-specific deviations in risk. The shape of the curves is similar for both sexes. The female cohort had a total of 19,608,727 exposures compared to 15,577,215 exposures in the male cohort. The RR was consistently higher among females across all age bands below 70 years, with the greatest disparity observed in the 35–40 age band. Above 70 years of age, RR values were almost identical between males and females.

Table 3 shows the AER and RR for the different MARS categories.

**TABLE 3:** AER and RR by MARS category.

MARS	Exposure	Number of deaths	AER (%)	RR (%)
Unknown	8,317 811	31,293	143.76	82.15
Low	22,549 919	111,311	185.18	105.82
Medium	1,877 840	14,219	180.21	102.98
High	1,902,892	22,837	155.76	89.01
Very high	537,480	13,187	228.55	130.6

MARS, medication adherence risk score.

AER, actual-to-expected ratio.

RR, relative risk.

The MARS “very high” category had the fewest exposures (n = 527,480) and the highest AER of 228.55%. The “low” and “medium” categories showed similar AERs of 185.18% and 180.21% respectively. The “high” category had the lowest AER (155.76%) among all known MARS categories.

**FIGURE 2:**
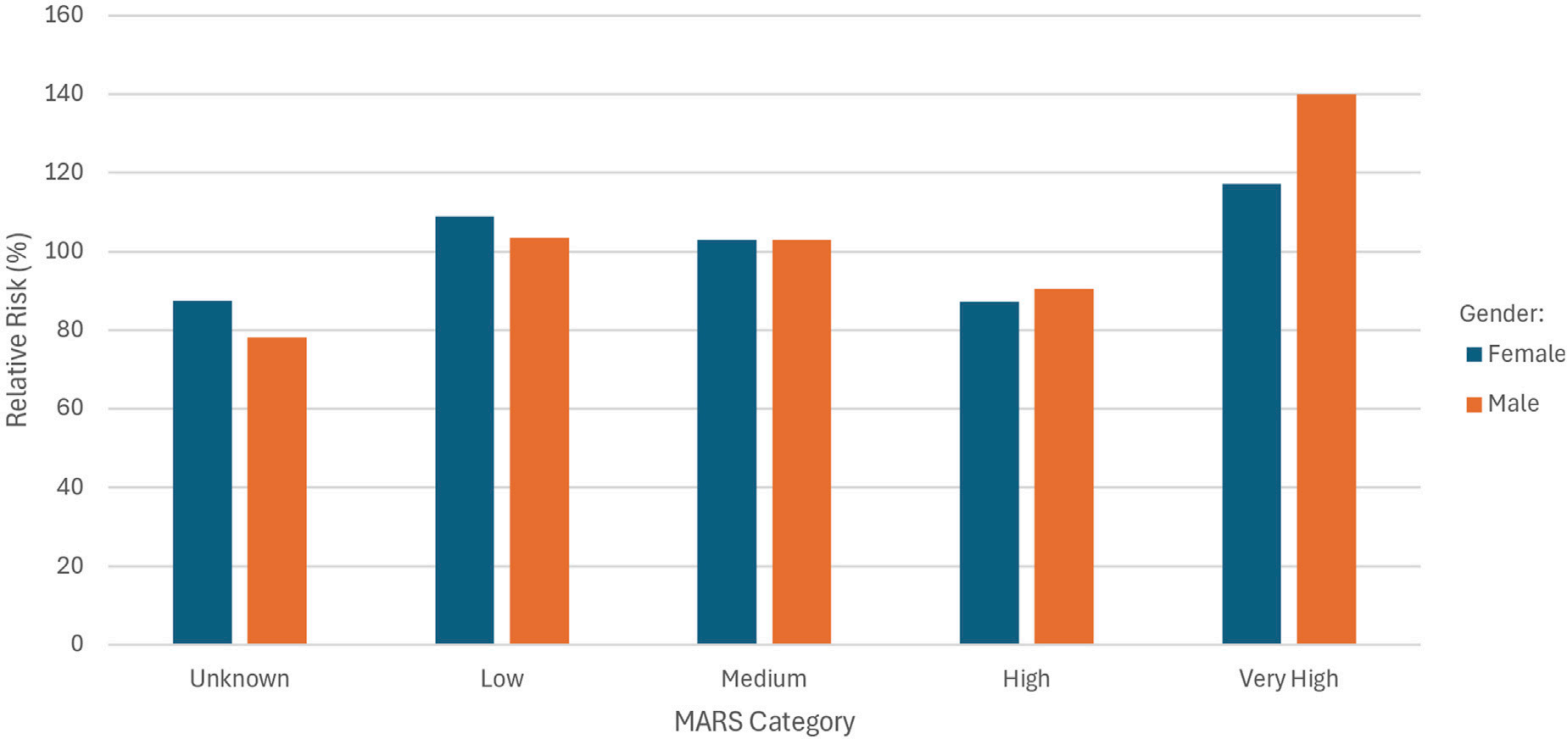
Relative risk by MARS category and gender.

Figure 2 shows the RR for the different MARS categories split by gender.

A higher RR was observed for females in the “unknown” and “low” MARS categories. In the “high” category, males showed a slightly elevated RR compared to females (90.42% vs. 87.22%), and a substantially higher RR in the “very high” category (139.88% males vs. 117.18% females).

**FIGURE 3:**
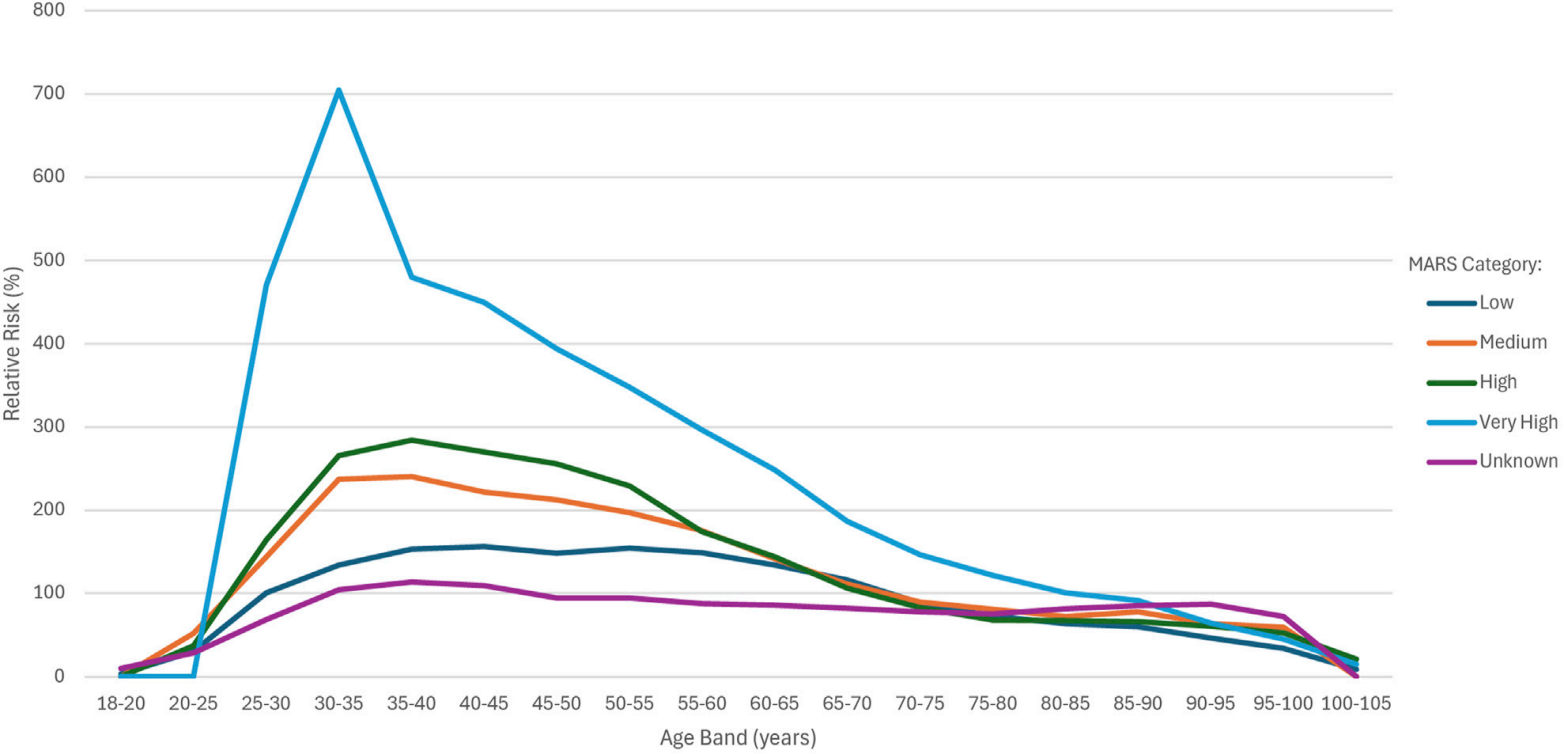
RR by MARS category and age band.

Figure 3 shows the RR for the MARS categories across the various age bands.

For the overall sample, increasing AER and RR did not correlate with increasing MARS categories. However, use of the MARS in addition to age band allowed for differentiation of risk within the 25 to 55 age bands, with a higher MARS score indicating a higher AER and RR. The RR for the very high category was substantially higher than all other groups up until the 85–90 age band, with a spike observed in the 30–35 age band. Between 55 and 60 years, the RR for the “medium” and “high” groups converged, whereas after 55 years, the RR for the high group was in line with the medium group and around 65 years, it dipped below the low group. The performance of the high group at older age bands explains why this category showed lower than expected RR when comparing MARS categories for the overall sample.

The time-to-event analysis showed a statistically significant difference (p < 0.001) in the mean number of months before death occurred between the different MARS categories (Table 4). The Schoenfeld plots indicated that the proportional hazards assumption was reasonably met.

**TABLE 4:** Mean time (in months) to death by MARS category.

MARS category	Mean number of months to death	p-value
Low	26.53	p < 0.001
Medium	8.93	p < 0.001
High	7.02	p < 0.001
Very high	6.92	p < 0.001

## 4 Discussion

### 4.1 Main findings

This study aimed to demonstrate that the MARS model can enhance risk stratification efforts by identifying individuals at increased risk of mortality related to non-adherence to chronic medication. The mortality experience of the study population was higher than the expected fully underwritten base (AER = 175%). The RR was greater than 100% in the 30 to 65 age bands for males and the 25 to 65 age bands for females. When considering the overall sample, increasing AER and RR did not correlate with increasing MARS categories. The very high MARS category had the lowest number of exposures (n = 527,480) and the highest AER (228.55%) and RR (130.6%). The low and medium groups had similar AERs (185.18% and 180.21%, respectively) and RRs (105.82% and 102.98%, respectively). The high group had the lowest AER (155.76%) and RR (89.01%) of known MARS categories. A similar trend in AER and RR across MARS categories was observed for both males and females. However, use of the MARS in addition to age band allowed for differentiation of risk within the 25 to 55 age bands, with a higher MARS score indicating a higher AER and RR.

It is expected that the fully underwritten base would have a better mortality experience than a non-underwritten cohort, given that the underwriting process tends to filter out higher-risk individuals. The study population was limited to people who had purchased from a Health Window partner pharmacy during the study period, with 83.33% of participants purchasing chronic medication. By comparison, the prevalence of chronic conditions such as hypertension and type 2 diabetes in the South African population is substantially lower (15% (Pheiffer et al., 2021) to 48% (Kandala et al., 2021)). This may explain the higher mortality experience in the sample compared to the fully underwritten base. For the purpose of this discussion, this will be referred to as the “pharmacy population effect”. Notably, the AERs greater than 200% were observed in the 30–65 age group. In the general population, prevalence of chronic conditions in younger age groups is even lower (3%–23%) (Roomaney et al., 2021), meaning that the pharmacy population effect is likely to be even more pronounced. There may have been other characteristics of the pharmacy population that were not observable in the data and may have contributed to this difference, remaining unknown to the researchers.

There were substantially more exposures in the female cohort than the male cohort (19,608,727 vs. 15,577,215, respectively). While the RR for females was higher than that of males, both had the same trend across age bands, where a spike across the 30–65 age bands was observed. This shows that gender did not have any impact on the age-specific deviations in risk observed in the overall sample. The RR for females and males was very similar for age bands >70 years. Differences in disease burden and mortality experience between genders are expected (Patwardhan et al., 2024). However, mortality experience of men in all age groups is typically higher than that of women (Zhao and Crimmins, 2022; Zarulli et al., 2021). In the general South African population, females have a lower incidence of death due to cardiovascular disease and chronic lung disease in younger age groups, but the trend reverses in older age groups (approx. 70–80 years) (Africa DoSS, 2023). It is possible that the pharmacy population effect may have been most pronounced in the younger female population, resulting in the higher RR observed.

The unknown MARS category had a lower AER and RR (143.76% and 82.15%, respectively) compared to any of the known MARS categories. Considering that individuals assigned to the “unknown” category had no observable presence of chronic disease and were likely healthier, with sporadic pharmacy use, this finding is not unexpected. When considering the known MARS categories, the likely expectation is that the RR would progressively increase from low to medium, high, and very high. While the highest AER and RR were observed in the very high category (228.55% and 130.6%, respectively) the sequence of the remaining categories followed a reversed trend (low AER = 185,18%, low RR = 105.82%; medium AER = 180.21%, medium RR = 102.98%; high AER = 155.76%, high RR = 89.01%). Gender did not have any impact on the trend observed across the known MARS categories.

The lack of a monotonic trend in the overall sample suggests that the MARS may not be a consistently reliable predictor, and its use as an independent risk factor should be approached with caution. It must be acknowledged that the dataset likely contained inherent limitations. Patients who access both public and private healthcare facilities, or who obtain medication from pharmacies within and outside of the Health Window network, may have been misclassified. Additionally, some patients may have been severely ill and unable to visit the pharmacy, resulting in drop-off and subsequent skewing of results. Furthermore, the discrepancy between script fills and the actual ingestion of medication is a critical consideration, as purchasing medication does not necessarily guarantee adherence to the prescribed dosing regimen. This phenomenon was demonstrated in a cohort study using mass spectrometry spot urinalysis to measure true adherence to anti-hypertensive medication (Curneen et al., 2023). Only 27.4% of participants demonstrated biochemical evidence of actual medication intake, despite 75.3% reporting adherence.

The discriminative capacity of the MARS model became evident through its application across the different age bands. Between age bands 25–55, the RR risk progressively increased from low to medium to high to very high. The degree of differentiation between the different groups was substantial and easily observed within these age bands and became less apparent from 55 years onwards. After 55 years, the RR for the high group was in line with the medium group and around 65 years, it dipped below the low group. The performance of the high group at older age bands explains why this category showed lower than expected RR when comparing MARS categories for the overall sample. The very high category was substantially higher than all other groups, up to 90 years of age. The only observable spike was in the very high category for age band 30–35 years. The unknown MARS category showed the lowest RR between 25 and 75 years of age and maintained a relatively flat shape. The method of calculating the MARS appeared to assign a score to those with higher risk and only leave those with the lowest risk unassigned. Given that MARS does not perform well in older populations, it cannot be considered as universally predictive. This relationship is not unexpected, as the relative risks of most major risk factors have been shown to decline with age (Mehta et al., 2019; Kaneko et al., 2025). A large 2006 meta-analysis showed that systolic blood pressure is an important modifiable risk factor for coronary heart disease, but in comparison with the effects of age itself, the effects of blood pressure are small (Collaboration, 2006).

When considering the practical application of a score like MARS, the value gained from improved risk prediction in younger age groups is likely to be greater than that of having improved risk prediction in older age groups. Risk profiles converge over 70 years of age, and the relative impact of most major risk factors diminishes, as evidenced by the declining hazard ratios for mortality (Mehta et al., 2019; Benzeval et al., 2011). Consequently, distinguishing between levels of risk becomes less actionable in older populations. Approximately 80% of the South African population is between the ages of 15 and 64 years, and the average life expectancy in South Africa is 61.5 years (Organization WH, 2025). These demographic realities further support the relevance and potential utility of the MARS model as a tool for risk stratification within a South African context.

The time-to-event analysis demonstrated a statistically significant association between MARS categories and survival time, with higher MARS scores linked to a notably shorter mean number of months to death (p < 0.001). The absolute survival times for the high and very high categories (7.02 and 6.92, respectively) support the conclusion that these groups did have an elevated risk profile. As the MARS is an objective measure calculated from dispensary transactional data alone, the clinical context of these groups is not known. However, the short survival times observed align with the high-risk classification indicated by the MARS. These results reinforce the MARS score’s value in predicting mortality outcomes.

### 4.2 Limitations

Limiting the study population to individuals actively purchasing medication from the Health Window pharmacy network introduced the “pharmacy population effect”. Other characteristics may have been present in this population that could not be observed in the data and remain unknown to the researchers. These include co-morbidities, socioeconomic status, and non-mortality outcomes. These factors are significant as they influence both health-seeking behaviour (including medication adherence) and mortality experience. This is highlighted as a key limitation. The researchers could not adjust for any of these factors, some of which may have had confounding effects. However, the size of the study population conveys a great degree of credibility to the results. Furthermore, the study aimed to demonstrate that the existing MARS is predictive of an increase in mortality risk, and this was demonstrated within the study population. The comparison to the fully underwritten population served as a benchmark against which to compare the results within the study population.

Prior to observing the results, the authors would have expected the AER and RR to increase progressively from low to medium, high, and very high. Although the MARS score was generally predictive, the AER and RR trend observed across the known MARS categories was not in line with expectations. This may have been due to certain limitations in the dataset, e.g., individuals who collect chronic medications from public facilities may not have been identified as chronic and may have been assigned to a lower MARS category. Furthermore, patients may have been misclassified due to moving between pharmacies within and outside of the Health Window network. Patients who are severely ill and unable to access the pharmacy may drop-out of the network and further affect the results.

The authors acknowledge the potential for left truncation bias, as individuals could only be included in the study if they survived long enough to have a dispensary transaction recorded. The study population was defined based on dispensary transactions from Health Window network pharmacies, meaning with at least one dispensary transaction during the study period were included. Individuals who died prior engaging with a relevant pharmacy could not be included. Given the nature of the score, this is unavoidable. Nevertheless, it must be noted that there may have been mortality experienced outside of this group that could not be accounted for.

The potential for immortal-time bias was also considered. This was resolved by including the “unknown” MARS category which represents individuals for which a score could not be assigned due to insufficient history. This approach helps to address the bias by ensuring that these individuals are still captured in the analysis, rather than being excluded or misclassified.

The study population was limited to individuals purchasing medication from private pharmacies. While this subgroup provides valuable insights into medication usage patterns and risk identification within the private healthcare sector, it represents only a small fraction of the broader South African population. Approximately 84% of South Africans are reliant on public healthcare (Ngobeni et al., 2020). As a result, demographics, health-seeking behaviours, socioeconomic status, and disease burden among private healthcare users may differ significantly from those accessing care through public facilities. There is substantial inequality between the private and public healthcare sectors in South Africa (Dell and Klopper, 2018; Ngene et al., 2023). The country is facing a quadruple burden of disease, with diseases and maternal conditions (associated with poverty and underdevelopment) accounting for 25% of years of life lost (Bradshaw et al., 2003). Rural South African populations experience a pronounced overlap of infectious and non-communicable disease epidemics (Wong et al., 2021). Furthermore, it has been noted that “postponement of care seeking and unmet need is concentrated among the socio-economically disadvantaged” within the South African context (Gordon et al., 2020). This limits the significance of the study as findings from private pharmacy data would require cautious extrapolation to the broader population.

The study period encompassed the years 2020 and 2021, during which the effects of the COVID-19 pandemic were most pronounced. This unprecedented public health crisis had a profound impact on global mortality patterns, with adult mortality rates increasing markedly (Collaborators, 2024). These effects were due to the COVID-19 infection, delayed care for other conditions, altered health-seeking behaviours, and economic factors (Zińczuk et al., 2023). In the South African context, the impact was heterogenous in nature, with black African females, aged 30–39 years, demonstrating the highest hospitalisation rates compared with other groups (Phaswana-Mafuya et al., 2021). Among Black African and Coloured populations, females exhibited higher COVID-19-relatred mortality than males (Phaswana-Mafuya et al., 2021). As a result, the spikes/anomalies seen in the younger age groups and the higher observed RR for females may be partially attributable to the direct and indirect effects of COVID-19. Consequently, the pandemic represents a confounding factor that could have influenced the study findings, limiting the generalisability of the results to non-pandemic periods.

While the novel predictive insight of MARS is promising, its proprietary calculation introduces an unavoidable limitation. Replication will require either disclosure of the calculation or development of an equivalent, transparent metric. All other analyses within this study are fully reproducible and documented for independent verification.

### 4.3 Future recommendations

To our knowledge, this study represents the first attempt to define and validate a mortality risk prediction model using pharmacy transactional data. While the initial results are promising, further research is warranted to refine the scoring algorithm and assess its applicability within specific use cases, e.g., healthcare prioritisation, and population health management. Further refinement of the “unknown” MARS category is recommended. A use case of particular relevance in resource-constrained settings (like South Africa) is improving cost efficiency in the delivery of care. By identifying individuals at increased risk of adverse health outcomes, such as hospitalisation and premature death, healthcare systems can allocate resources more strategically. This enables more targeted interventions and can reduce high-cost events and avoidable utilisation of healthcare services.

Future research that includes both public and private healthcare data would help to provide a more comprehensive understanding of risk and the applicability of an adherence-based score across the South African population. Furthermore, research spanning longer timeframes or data following the COVID-19 pandemic is encouraged.

In future, research aims could be expanded on to include more advanced statistical modelling and proprietary tool validation. This could include a Poisson Generalized Linear Model to confirm whether the MARS categories are statistically significant predictors of mortality rate, independent of the baseline actuarial model (Roccetti and Cacciapuoti, 2025), and a Kolmogorov-Smirnov Test to provide further non-parametric support for the score (Roccetti and Cacciapuoti, 2025). Furthermore, the authors recommend running a prospective study to further validate this work, in light of the limitation created by missing records.

## 5 Conclusion

While the absence of a monotonic trend limits the generalisability of the MARS, it was shown to be predictive of mortality risk when used in conjunction with age. The results of this study show that the MARS score was able to differentiate mortality risk within the 25 to 55 age range. It is most effective within this cohort. Where a MARS score could not be assigned to an individual, this reflected the lowest mortality risk. The standard fully underwritten model underestimated the number of deaths within this pharmacy population. The MARS model identified the increase in mortality risk within this population, which would otherwise have been missed. The time-to-event analysis showed a significant inverse relationship between MARS category and survival time.

The MARS is an objective and replicable score that can be calculated for the South African population utilising private healthcare services (approximately 16% of the total population (Ngobeni et al., 2020)). Further research should focus on refining and improving the score and its performance in specific contexts, as well as understanding its applicability within the public healthcare sector.

The researchers support the implementation of such a model at scale and advocate for its integration into healthcare planning and policy frameworks, which could inform both clinical decision-making and broader population health strategies.

## Data Availability

The raw data supporting the conclusions of this article will be made available by the authors, without undue reservation.
