# Supplementary material for: Beyond prescriptions: chronic medication adherence predicts mortality risk in a large-scale cohort study
Source: Front Pharmacol. 2025 Nov 25;16:1701588. doi: 10.3389/fphar.2025.1701588 (PMC12685934; doi:10.3389/fphar.2025.1701588)
Supplement: Supplementary file 1 [file DataSheet2.pdf]

Supplementary File 2: Inclusion and exclusion criteria for sample selection

Inclusion criteria for sample selection

**Inclusion Criterion**	**Justification**	**Methodology**
Valid South African identity number.	Certain fields may be captured incorrectly on pharmacy dispensing systems. This can result in identity numbers containing too many digits, too few digits or text characters. Invalid identity numbers introduce the risk of being unable to match the individual to other datasets.	Only individuals with identity numbers containing 13 numerical digits were included.
At least one transaction from a partner pharmacy from January 2017 to May 2022.	Limiting the study to individuals with transactions during the exposure and outcome measurement period ensured they were active consumers within the pharmacy network. Customers who entered the pharmacy network after the analysis period were irrelevant to the study.	Individuals with no transactions during this time period were removed from the dataset used in the study.

<u>Exclusion criteria for sample selection</u>

**Exclusion Criterion**	**Justification**	**Methodology**
Participant age was calculated as a negative number.	Certain fields may be captured incorrectly on pharmacy dispensing systems. Occasionally, an incorrect date of birth or identity number was entered, resulting in the participant's age being reflected as a negative number. This made up approximately 0.2% of the total records.	Only individuals aged >0 years were included.

Pharmacy transactions occurred after the indicated date of death.	Certain fields may be captured incorrectly on pharmacy dispensing systems. Occasionally medication is allocated to the wrong individual within a pharmacy's family profile. This can result in a medication purchase being allocated to an already deceased individual. This was less than 0.1% of the total records.	Individuals with transactions occurring after the indicated date of death were removed from the sample.
