# Supplementary material for: Beyond prescriptions: chronic medication adherence predicts mortality risk in a large-scale cohort study
Source: Front Pharmacol. 2025 Nov 25;16:1701588. doi: 10.3389/fphar.2025.1701588 (PMC12685934; doi:10.3389/fphar.2025.1701588)
Supplement: Supplementary file 2 [file DataSheet1.pdf]

Supplementary File 1: Chronic Medication Adherence Score and Medication Adherence Risk Score

1. Chronic medication adherence score:

Pharmaceutical products within the Health Window database were flagged as "chronic" if the product was typically used on a long-term or repeatable basis. For all pharmaceutical molecules at Anatomical Therapeutic Chemical (ATC) level 5 flagged as “chronic”, a dispense frequency (DF) is calculated as the number of dispenses over the last 12 months divided by 12. To include a molecule in the DF calculation for a particular patient, the patient must have used a product with this ATC level 5 within the first month of the 12-month period, or within the 6 months before the 12-month period for which the dispense frequency is calculated. The dispense frequency per molecule is calculated. The overall average chronic, molecule-level adherence is then calculated for an individual patient.

The chronic medication adherence score may have some inherent limitations in predicting mortality risk. Firstly, a chronic flag is applied to any product typically used repeatedly. For example, the oral contraceptive pill, while not indicative of a health condition likely to increase risk of mortality, would receive a chronic flag in the company database as a patient can be expected to use this on a long-term basis. Secondly, the score does not consider differences in risk attributed to expected disease progression in individuals with a longer-standing diagnosis.

2. Medication Adherence Risk Score:

The MARS was developed to address some of the potential limitations in the chronic medication adherence score’s ability to predict mortality risk. The MARS is informed using the following factors:

- Number of unique risk-labelled Monthly Index of Medical Specialities (MIMS) classes from which a chronic product has been purchased at least 3 times in the last 24 months
- Chronic molecule-level adherence rate for each applicable MIMS class
- Duration of use of treatment within each MIMS class

This calculation of this score is limited to the following MIMS classes which the researcher believed to be the most indicative of overall health risk:

MIMS description	MIMS code
Anti-hypertensives	7.3
Cardiac glycosides	7.1.1
Anti-diabetic agents	19.1

Hipolipidaemic agents	7.7
Anti-anginal agents	7.4
Anti-arrythmics	7.2
Platelet aggregation inhibitors	8.4
Cytostatics	23.0.0
Anti-viral agents	18.12.0
Anti-asthmatics	10.4
Bronchodilators	10.2
Osteoporosis	4.7
Thyroid	19.3
Anti-depressants	1.4
Anti-psychotics	1.5
Anti-epileptics	1.6.0
Biologicals	26.0.0

For each MIMS class where a chronic product has been purchased at least 3 times in the last 24 months, a score is allocated according to the duration of use of chronic products and the chronic medication adherence score for molecules within that class:

- The “Overall Chronic Adherence %” is a molecule-level dispense frequency calculated within each MIMS class and converted to a percentage
- The “Chronic Status Duration” is the difference between today’s date and the date of the first chronic dispense within the MIMS class

Overall Chronic Adherence%	Score	Chronic Status Duration	Score
>80%	0	Less than 2 years	1
60-80%	2	2-4 years	2
40-60%	4	4-6 years	3
20-40%	6	More than 6 years	4
<20%	8		

A total score is allocated to each MIMS class, for example:

MIMS class	Chronic adherence score	Chronic duration score	Total score (sum of chronic adherence and chronic duration score)
Anti-hypertensives	4	1	5
Anti-asthmatics	6	3	9

The MIMS classes are weighted differently, depending on their expected contribution to morbidity and mortality risk:

Higher risk	Moderate risk	Lower risk
Anti-hypertensives 7.3	Anti-viral agents 18.12.0	Osteoporosis 4.7
Cardiac glycosides 7.1.1	Anti-asthmatics 10.4	Thyroid 19.3
Anti-diabetic agents 19.1	Bronchodilators 10.2	Anti-depressants 1.4
Hipolipidaemic agents 7.7		Anti-psychotics 1.5

Anti-anginal agents 7.4		Anti-epileptics 1.6.0
Anti-arrythmics 7.2		Biologicals 26.0.0
Platelet aggregation inhibitors 8.4		
Cytostatics 23.0.0		

Scores for “higher risk” MIMS classes are multiplied by a factor of 1.

Scores for “moderate risk” MIMS classes are multiplied by a factor of 1/2 (0,5).

Scores for “lower risk” MIMS classes are multiplied by a factor of 1/7 (0,1428).

The sum of all the weighted scores would result in a final overall Medication Adherence Risk Score for the individual. A score would then be classified into one of the following groups:

- Very high
- High
- Medium
- Low

In order to establish the cut-off points for the various groups, a score was calculated for every individual within the company database. The 'very high’ group was reserved for the 1% of the population with the highest scores. The limits for the other groups were determined by the researcher using the process shown below:

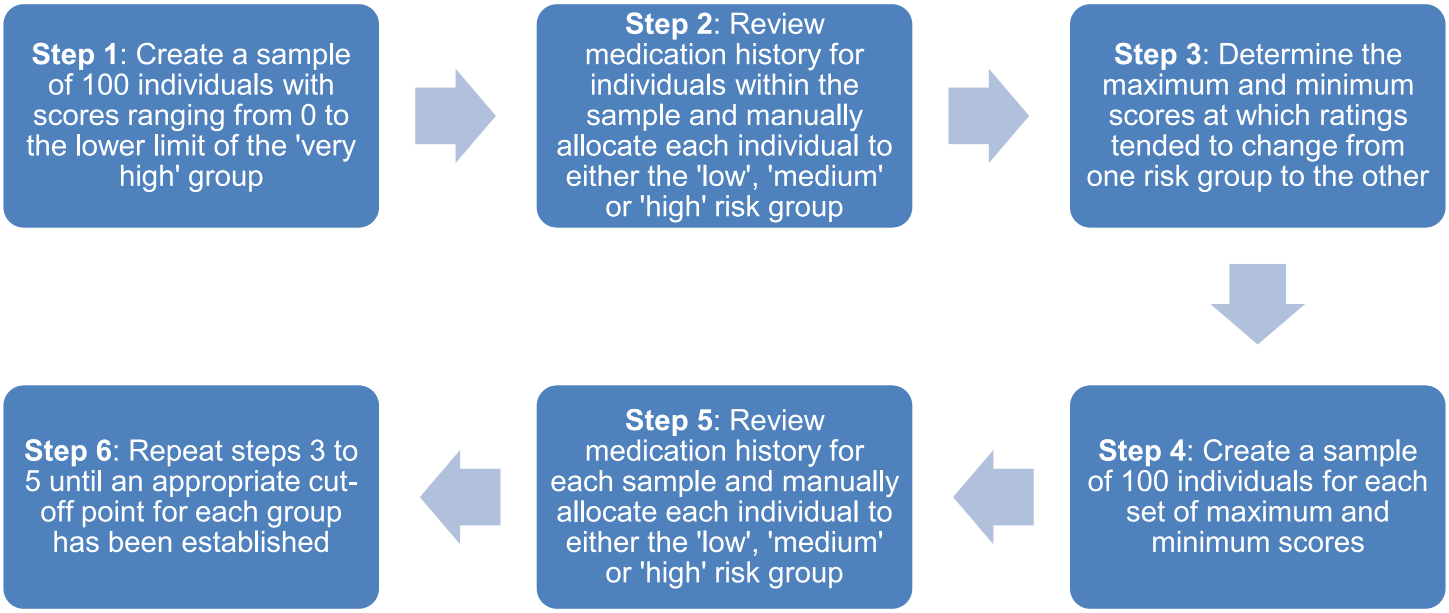

This process resulted in the groups being defined as follows:

- Very high = the top 1% of the population (not a fixed score range)
- High = score between 3,05 and the minimum of the very high population (score range varies as the threshold for the very high-risk group varies)
- Medium = fixed score between 0,11 and 3,05 (excluding 3,05)
- Low = fixed score between 0 and 0,11 (excluding 0,11)
